# Insulin Modulates Cytokine Release, Collagen and Mucus Secretion in Lung Remodeling of Allergic Diabetic Mice

**DOI:** 10.3389/fimmu.2017.00633

**Published:** 2017-06-09

**Authors:** Sabrina S. Ferreira, Fernanda P. B. Nunes, Felipe B. Casagrande, Joilson O. Martins

**Affiliations:** ^1^Laboratory of Immunoendocrinology, Department of Clinical and Toxicological Analyses, Faculty of Pharmaceutical Sciences of University São Paulo (FCF/USP), São Paulo, Brazil

**Keywords:** asthma, diabetes mellitus, insulin, remodeling, eosinophils, collagen, mucus, lung

## Abstract

**Introduction:**

The role of insulin in lung remodeling in a model of asthma in healthy and diabetic mice was evaluated.

**Material and methods:**

Diabetic male BALB/c mice (alloxan, 50 mg/kg, intravenous) and controls were sensitized by subcutaneous (s.c.) injection of ovalbumin (OA, 20 µg) in aluminum hydroxide (Al(OH)_3_, 2 mg) 10 days after the alloxan injection and received the same dose 12 days later. Six days after the last sensitization, animals were nebulized with OA solution for 7 days. The first set of diabetic and control mice received 2 and 1 IU, respectively, of s.c. neutral protamine Hagedorn (NPH) insulin and were analyzed 8 h later. The second set of diabetic and control mice received 2 and 1 IU, respectively, of insulin 12 h before the OA challenge and half doses of insulin 2 h before each the seven OA challenges. Twenty-four hours after the last challenge, the following analyses were performed: (a) quantification of the cells in the bronchoalveolar lavage fluid (BALF), the white cell count, and blood glucose; (b) morphological analysis of lung tissues by hematoxylin and eosin staining; (c) quantification of collagen deposition in lung tissues and mucus by morphometric analysis of histological sections stained with Masson’s trichrome and periodic acid-Schiff (PAS), respectively; and (d) quantification of the cytokine concentrations (IL-4, IL-5, and IL-13) in the BALF supernatant.

**Results:**

Compared to controls, diabetic mice had significantly reduced inflammatory cells (81%) in the BALF, no eosinophils in the BALF and peripheral blood and reduced collagen deposition and mucus in the lungs. BALF concentrations of IL-4 (48%) and IL-5 (31%) decreased and IL-13 was absent. A single dose of insulin restored peripheral blood eosinophils and BALF mononuclear cells but not BALF eosinophils, collagen deposition, and mucus levels. However, multiple doses of insulin restored both total cells and eosinophils in the BALF and peripheral blood, BALF cytokines, and collagen deposition and mucus secretion into the lungs.

**Conclusion:**

The results suggest that insulin modulates the production/release of cytokines, cell migration, deposition of collagen, and mucus secretion in lung remodeling of a mouse model of asthma.

## Introduction

Asthma is characterized by chronic inflammation of the airways and is related to exposure to allergens, infections and other factors (1). The inflammatory process in allergic asthma is predominantly characterized by increased number of eosinophils, activated mast cells, and Th2 lymphocytes (2, 3). Airway inflammation, airflow obstruction, and bronchial hyperresponsiveness are characteristic features of asthma (4–6). Airway remodeling is the result of various structural changes in the airways (7, 8). Asthma affects approximately 300 million people worldwide (9), while overall prevalence in Brazil is 10–25% (1).

Experimental and clinical studies have indicated that the inflammatory response is impaired in diabetic patients. Triggering of diabetes mellitus in asthmatic patients resulted in an improvement in the asthmatic condition and treatment with insulin restored asthma symptoms (10, 11). Insulin has been shown to modulate inflammatory components of asthmatic reactions (12, 13). Previous studies demonstrated that alloxan-induced diabetic rats present substantially reduced mast cell degranulation upon antigen challenge. Treatment of diabetic rats with insulin restored the number of degranulated mast cells, histamine release, and airway reactivity to ovalbumin (OA) (13). In addition, animals rendered diabetic by alloxan injection exhibited reduced pulmonary inflammatory infiltrate. Insulin treatment restored this condition, suggesting a major role of insulin in asthma (14). In a similar model of asthma, insulin was shown to modulate the production of cytokines, such as TNF-α and IL-1β, along with expression of adhesion molecules (P- and E-selectins) and neutrophil migration into the lungs (12). We thus examined whether insulin modulates lung remodeling in a murine model of allergic lung inflammation. This study aimed to evaluate the role of insulin in lung remodeling of a model of asthma in healthy and diabetic mice.

## Materials and Methods

### Animals

We used specific pathogen-free male BALB/c mice, 8–12 weeks of age, weighing approximately 20–25 g at the beginning of the experiments. The animals were maintained at 22°C under a 12 h light–dark cycle and were allowed access to food and water *ad libitum* throughout the observation period. This study was carried out in strict accordance with the principles and guidelines adopted by the Brazilian National Council for the Control of Animal Experimentation (CONCEA) and approved by the Ethical Committee on Animal Use (CEUA) of the Faculty of Pharmaceutical Sciences (FCF) of University São Paulo (Permit Number: CEUA/FCF/340). All surgical procedures were performed under ketamine/xylazine anesthesia, and all measures were taken to minimize suffering.

### Induction of Diabetes Mellitus

Diabetes mellitus was induced by intravenous injection of alloxan monohydrate (50 mg/kg; Sigma Chemical Co., St. Louis, MO, USA) dissolved in physiologic saline (SAL, 0.9% NaCl). Control mice were injected with physiologic SAL only. After 10 days, the presence of diabetes was verified by blood glucose concentrations higher than 300 mg/dL, which were determined with a blood glucose monitor (Accu-Chek Advantage II, Roche Diagnostica, São Paulo, São Paulo, Brazil), in blood samples obtained from mouse tails (15).

### Induction of Allergic Asthma

Mice were sensitized on days 10 and 22 by intraperitoneal (i.p.) injection containing 20 µg of OA (Sigma, USA) and 2 mg of aluminum hydroxide [Al(OH_3_); Reheis Inc., USA] in PBS to a total volume of 0.2 mL. Sensitized and control mice were challenged by multiple exposures to aerosol (5% OA in PBS) from an ultrasonic nebulizer (ICEL US-800, São Paulo, Brazil), delivering particles of 0.5–10 µm diameter at approximately 0.75 cc/min for 30 min. Challenges were performed daily for 7 days (28–33 and 35). The experiments were performed 24 h after the last challenge (16).

### Insulin Treatment

**Figure 1:**
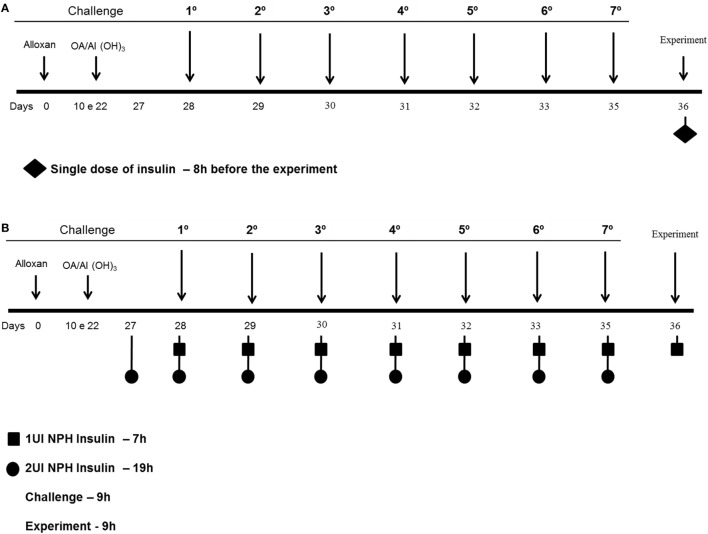
**Experimental protocols: mice were rendered diabetic by the injection of alloxan (50 mg/kg, i.v.)**. Ten days after, they were sensitized 2× intraperitoneal with OA and Al(OH_3_) and then subjected to aerosol antigen challenges (7 days) with OA (5%, w/v in PBS) or PBS alone. **(A)** The first set of diabetic and control mice received 2 and 1 IU, respectively, of NPH insulin subcutaneously 24 h after the last challenge, and the analyses were performed 8 h after the insulin treatment. **(B)** The second set of diabetic and control mice received 2 and 1 IU, respectively, of insulin subcutaneously 12 h before the OA challenges (07:00 p.m.) and half doses (07:00 a.m.) of insulin 2 h before each of the 7 OA challenges; 24 h after the last challenge, blood, lungs, and BALF were collected for further analysis.

Diabetic and control mice were divided into two groups according to the different insulin treatments. The first set of diabetic and control mice received 2 and 1 IU, respectively, of neutral protamine Hagedorn (NPH; Eli Lilly, São Paulo, São Paulo, Brazil) insulin subcutaneously 24 h after the last challenge, and the analyses were performed 8 h after the insulin treatment (Figure 1A) (14). The second set of diabetic and control mice received 2 and 1 IU, respectively, of insulin subcutaneously 12 h before the OA challenges (07:00 p.m.) and half doses (07:00 a.m.) of insulin 2 h before each of the 7 OA challenges (Figure 1B). After 24 h, blood, lungs and bronchoalveolar lavage fluid (BALF) were collected for further analysis (17).

### Kinetics of Glucose with Insulin Treatment

Glucose measurements for a kinetic curve were performed to determine when the challenges should be performed after insulin treatment. Diabetic and control mice received 2 and 1 IU, respectively, of NPH insulin subcutaneously, and glucose levels were determined at 1, 2, 3, 6, and 8 h after the insulin treatment.

### Bronchoalveolar Lavage

Mice were euthanized by a lethal dose of ketamine hydrochloride (90 mg/kg) and xylazine hydrochloride (10 mg/kg). The trachea was cannulated with polyethylene tubing (24 G3/4). The lungs were then lavaged by instillation of 1 mL of PBS (pH 7.4) three times for total volume of 3 mL. The BALF was centrifuged at 1,500 rpm for 10 min, and supernatant was frozen at −70°C for later cytokine measurements. Pelleted cells were collected and resuspended in 1 mL of PBS. The total number of cells was determined using a Neubauer chamber, whereas a differential count was obtained after cells from the BALF were centrifuged.

### Quantification of Cytokines in the BALF

The level of cytokines (IL-1β, IL-4, IL-5, IL-10, and IL-13) was measured in BALF supernatant samples by enzyme-linked immunosorbent assay (ELISA), using commercial kits (R&D Systems, Inc., Minneapolis, MN, USA). Assays were performed according to the manufacturer’s manual.

### Hematological Parameters

Samples of blood collected by the intracardiac route were used for determination of the cell numbers. The total value was determined with an automated hematology counter (ABC Vet—Horiba ABX). Blood smears were stained with Rosenfeld. A total of 100 cells were counted using a conventional optic microscope (Leica Microsystems, Wetzlar, Germany).

### Lung Morphology Analysis

For histological analysis, after collection of BALF, the lungs were removed and fixed in 10% formaldehyde solution, processed, and embedded in paraffin. Sections of 5 µm were cut, mounted on slides, and stained with hematoxylin and eosin (H/E) for observation of airway morphology; Masson’s trichrome staining was performed for observation of collagen deposition around the airways. The collagen fibers appeared blue, the nuclei were black and the rest of the tissue (muscle, cytoplasm) stained red. Periodic acid-Schiff (PAS) histochemical staining was used to characterize the glycoprotein component of goblet cells in the respiratory epithelium (evaluation of mucus production). Slides containing the tissue were observed under a light microscope (Nikon Eclipse 80i, Tokyo, Japan) and photographed using the NIS-Elements AR 3.1 (SP3 build634) imaging software (Nikon).

### Mucus Deposition Quantification and Collagen

After morphological analysis, the positive area was measured (μm^2^). The maximum number of bronchioles per slide was determined. A measure of similar diameters was standardized to rule out the influence of bronchiole gage. The results are expressed as the mean total/diameter of area for each animal.

### Statistical Analyses

Data were processed and analyzed by analysis of variance (ANOVA) or an unpaired *t*-test using GraphPad Prism (version 6.0 for Windows, GraphPad Software, La Jolla, CA, USA). A two-tailed *p*-value with a 95% confidence interval was acquired. Data are represented as the mean ± standard error of the mean (SEM). *p*-Values <0.05 were considered significant.

## Results

### Body Weight Gain, Blood Glucose Levels, Insulin Treatment

Diabetes was induced by alloxan injection, and after 10 days, blood glucose levels and body weight gain were measured. Relative to controls, alloxan-treated diabetic mice exhibited a significant reduction in body weight gain (mean ± SEM; control, 1.05 g ± 0.32 g, *n* = 20; diabetic, −2.53 g ± 0.74 g, *n* = 20; *p* < 0.001) during the 10-day period and sharply elevated blood glucose levels (control, 123.14 ± 3.64 mg/dL, *n* = 20; diabetic, 559.5 ± 14.15 mg/dL, *n* = 20; *p* < 0.0001). Data collected on the 36th day showed that diabetic animals maintained insulinopenic characteristics throughout the experimental period. Diabetic animals that received daily doses of insulin for 9 days showed increases in body weight compared to that of the untreated diabetic mice. The weight increase was 50% compared to that of the non-diabetic asthmatic animals. A single dose of insulin did not rescue the body weight of the animals. Regarding the blood glucose, diabetic mice (both treated with 16 doses of insulin and not treated) showed high plasma glucose concentrations on the 36th day (Table 1).

**Table 1:** **General characteristics of the mice**.

Groups	*n*	Body weight gain (g)	Blood glucose (mg/dL)
Non-diabetic (10 days)	20	1.05 ± 0.32	123.14 ± 3.64
Diabetic (10 days)	20	−2.53 ± 0.74^#^	559.25 ± 14.15^+^

Non-diabetic + sensitized + saline (SAL) challenged (36 days)	5	1.58 ± 0.79	148.3 ± 8.83

Non-diabetic + sensitized + ovalbumin (OA) challenged (36 days)	5	1.61 ± 0.64	125 ± 5.88

Non-diabetic + sensitized + OA challenged + I1 (36 days)	5	1.78 ± 0.75	157.7 ± 7.77

Non-diabetic + sensitized + OA challenged + I16 (36 days)	5	1.78 ± 0.75	133.9 ± 8.86

Diabetic + sensitized + SAL challenged (36 days)	5	−3.9 ± 1.8	570 ± 15

Diabetic + sensitized + OA challenged (36 days)	5	−6.10 ± 2.05	551 ± 20

Diabetic + sensitized + OA challenged + I1 (36 days)	5	−5.88 ± 1.09	558.30 ± 15.79

Diabetic + sensitized + OA challenged + I16 (36 days)	5	2.88 ± 0.88^•^	467 ± 27.5

*Mice were rendered diabetic by the injection of alloxan (50 mg/kg, i.v.). Ten days after, non-diabetic and diabetic mice were subjected to two sensitization procedures (20 µg OA + Al[OH_3_]); intraperitoneal route. Twelve days after, non-diabetic and diabetic mice were subjected to OA (1% OA for 30 min) or SAL challenge (7 days). Insulin (single-dose 2 IU/diabetic mice or 1 IU/non-diabetic mice, 8 h before the experiment, s.c. 16 doses 2 IU/diabetic mice 12 h before challenge, 1 IU/diabetic mice 2 h before challenge or 1 IU/non-diabetic mice, 2 h before challenge, s.c.). Blood glucose levels were determined before insulin treatment and the experiment. Values are shown as the mean ± SEM*.

^#^*p < 0.001 compared with non-diabetic mice*.

^+^*p < 0.001 compared with non-diabetic mice*.

^•^*p < 0.05 compared with diabetic mice treated with a single dose of insulin*.

*Differences among the initial groups (diabetic or not) were analyzed using Student’s *t*-test. Differences among the groups were tested with one-way analysis of variance followed by Turkey’s *post hoc* test. A *p*-value <0.05 was considered statistically significant (GraphPad Prism version 6.0 for Windows, GraphPad Software, La Jolla, CA, USA)*.

### Kinetics of Blood Glucose in Diabetic and Control Animals

Diabetic animals received a single dose of NPH insulin (2 UI). One hour after insulin therapy, the blood glucose of the animals was reduced to approximately half the original values, and 2 h after treatment, it increased again, as the animals displayed hyperglycemia. Similar results were observed in the control animals (1 UI). In both groups, blood glucose values were similar to pretreatment values after 6 h of insulin administration, indicating that this dose was not sufficient to normalize glycemia. Thus, we believe that the effects observed in insulin-treated mice are primarily due to the increased levels of insulin rather than to normalization of glycemia (Table 2).

**Table 2:** **Glucose kinetics**.

Animals	Blood glucose					
	Before insulin	After insulin				
		1 h	2 h	3 h	6 h	8 h
Diabetic	500 ± 19	285 ± 38*	372 ± 20	342 ± 95	501 ± 44	568 ± 15
Non-diabetic	149 ± 11	68 ± 8^#^	67 ± 2	62 ± 2	134 ± 13	153 ± 15

*The animals were treated with alloxan or saline as described above. Diabetic animals were administered 2 UI NPH insulin, and control animals received 1 UI NPH insulin. Blood samples were obtained from tail samples, and glucose was assessed with a glucometer (Accu-Check Active—Roche Diagnosis^®^). Values represent the mean ± SEM of blood glucose (*n* = 6 animals per group)*.

*p < 0.05, significantly different from pretreatment blood glucose levels with insulin.

*^#^p < 0.05, significantly different from pretreatment blood glucose levels with insulin*.

*Differences among the initial groups (diabetic or not) before and after insulin treatment were analyzed using Student’s *t*-test*.

### Effect of insulin on peripheral blood cells and their migration

Relative to control (non-diabetic) OA-challenged mice, diabetic mice showed reduced leukocyte counts in the peripheral blood after OA challenge, including a reduction in the number of eosinophils. Treatment with single dose of NPH insulin 8 h before the experiment restored the impaired eosinophils to 46%. In addition, multiple doses of insulin restored the impaired eosinophils to 66%.

Relative to control (non-diabetic) OA-challenged mice, leukocyte counts in the BALF of diabetic mice were reduced after OA challenge due to an 83% reduction in the number of mononuclear cells, without the presence of eosinophils. Treatment with a single dose of insulin 8 h before the experiment restored the impaired cell migration observed in diabetic mice to values attained in control non-diabetic mice, but it did not restore the eosinophil migration. However, multiple doses of NPH insulin completely restored both total cell migration and the eosinophils in the BALF.

**Figure 2:**
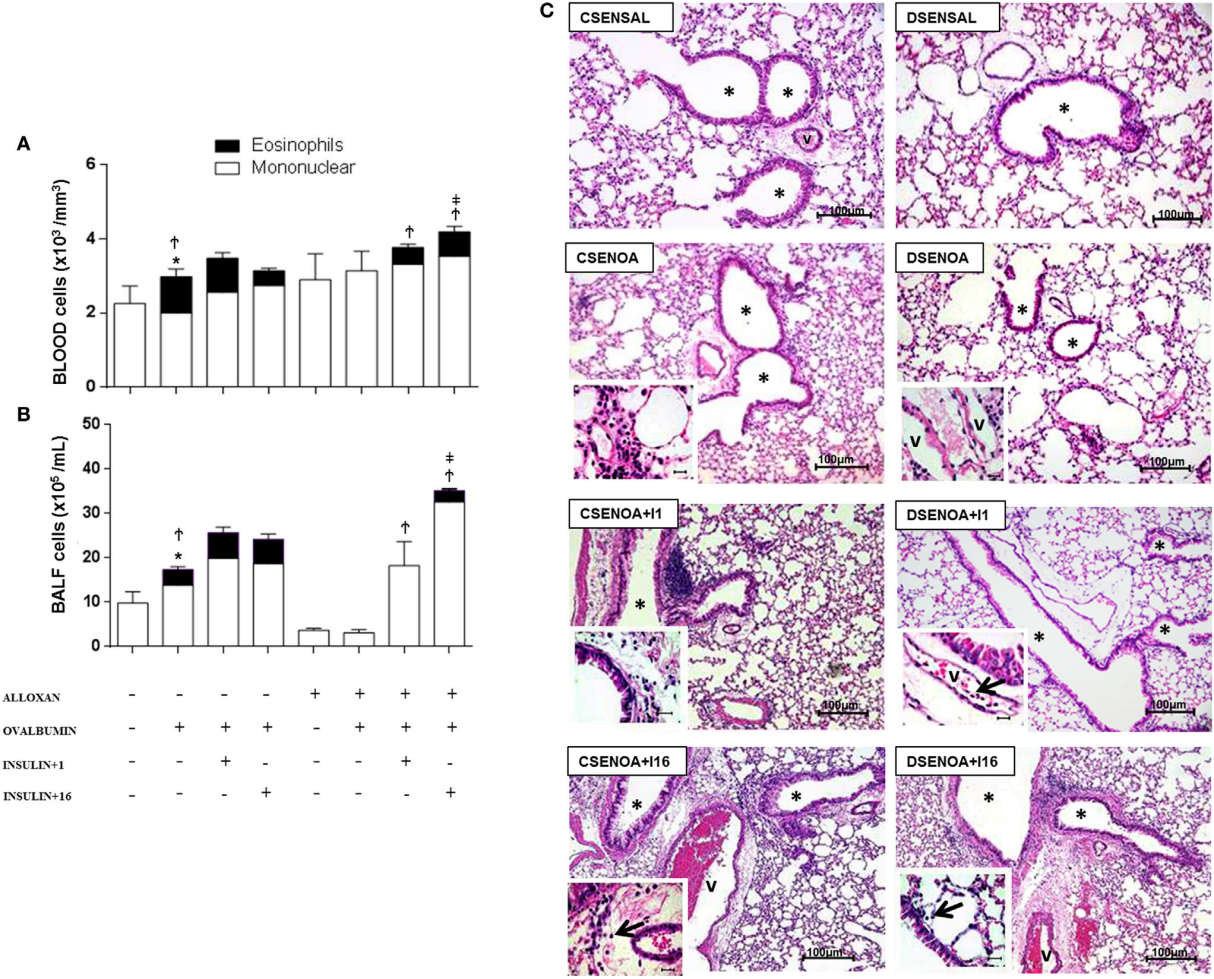
**Role of insulin on peripheral blood cells and their migration**. **(A)** Twenty-4 hours after the last challenge in each of the groups, peripheral blood was collected to count cells; **(B)** bronchoalveolar lavage fluid was assessed 24 h thereafter. **(C)** The microphotographs of lung tissue were obtained from control non-diabetic mice sensitized and instilled with saline (CSENSAL) or sensitized and instilled with ovalbumin (OA) (CSENOA) and treated with single-dose insulin (CSENOA + I1) or treated with multiple doses of insulin (CSENOA + I16); diabetic mice sensitized and instilled with saline (DSENSAL), or sensitized and instilled with OA (DESENOA) and treated with single-dose insulin (DSENOA + I1) or treated with multiple doses of insulin (DSENOA + I16). * = bronchus; v = blood vessel; arrow: eosinophil (bars = 100 µm) H/E. Values are shown as the mean ± SEM. *p < 0.01 comparing OA-challenged with saline-challenged group. ^Ϯ^*p* < 0.01 comparing OA-challenged with the diabetic OA-challenged group. ^‡^*p* < 0.001 comparing diabetic OA-challenged treatment to single-dose insulin. Data are representative of five animals per experimental group. Differences among the groups were tested with one-way analysis of variance followed by Tukey’s *post hoc* test. A *p*-value <0.05 was considered statistically significant (GraphPad Prism version 6.0 for Windows, GraphPad Software, La Jolla, CA, USA).

The morphometric analysis of lung parenchyma showed that compared to controls, the allergic reaction induced cell infiltration around blood vessels and in the lungs 24 h after the last challenge. However, diabetic mice exhibited reduced cell infiltration around the vessels and into the lungs. Treatment with a single dose of insulin 8 h before the experiment restored the amount of cells inside the blood vessels. In addition, multiple doses of insulin restored the cell migration levels around blood vessels and into the lungs (Figure 2).

### Effect of Insulin on Cytokine Concentrations

**Figure 3:**
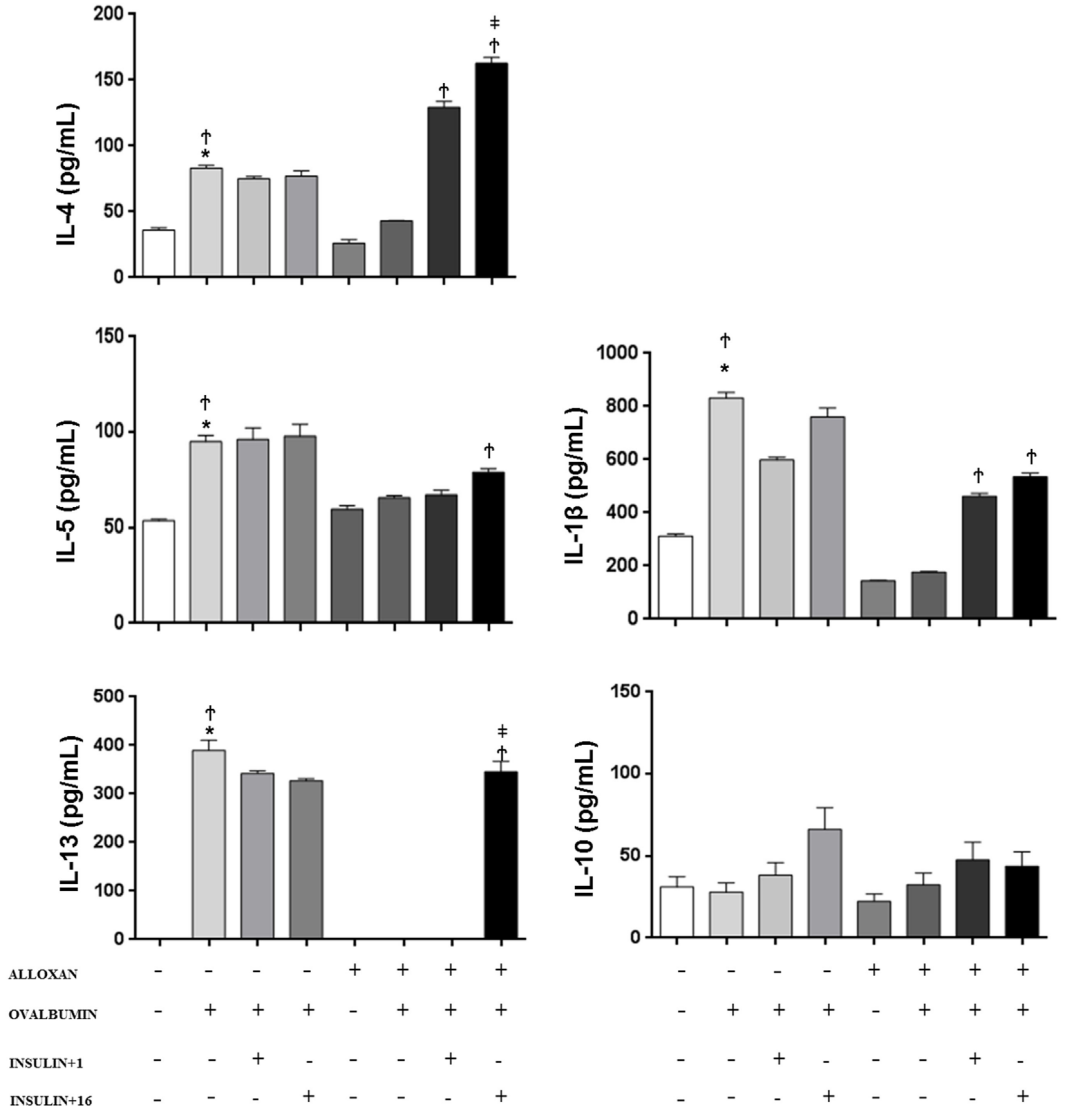
**Effect of insulin on cytokine concentrations**. Bronchoalveolar lavage fluid was analyzed 24 h after ovalbumin (OA) sensitization of non-diabetic and diabetic mice 24 h after the last OA (experimental) or saline (control) instillation. Values are shown as the mean ± SEM. *p < 0.01 comparing OA-challenged with the saline-challenged group; ^Ϯ^*p* < 0.01 comparing OA-challenged with the diabetic OA-challenged group. ^‡^*p* < 0.05 comparing diabetic OA-challenged treatment to single-dose insulin. Differences among the groups were tested with one-way analysis of variance followed by Tukey’s *post hoc* test. A *p*-value <0.05 was considered statistically significant (GraphPad Prism version 6.0 for Windows, GraphPad Software, La Jolla, CA, USA).

In the BALF of non-diabetic mice, we observed an increase in the concentration of IL-1β (2.6-fold), IL-4 (2.3-fold), IL-5 (1.8-fold), and IL-13 after the OA challenge. In contrast, diabetic OA-challenged mice presented a reduction in the levels of IL-1β (21%), IL-4 (51%), and IL-5 (68%), and IL-13 was not detected in the BALF. Treatment of diabetic mice with a single dose of insulin 8 h before the experiment restored BALF IL-4 and IL5 levels. Furthermore, multiple doses of insulin completely restored the IL-13 levels. The IL-10 levels were similar in all the studied groups, and there was no statistical difference between the groups (Figure 3).

### Collagen Secretion: Role of Insulin

**Figure 4:**
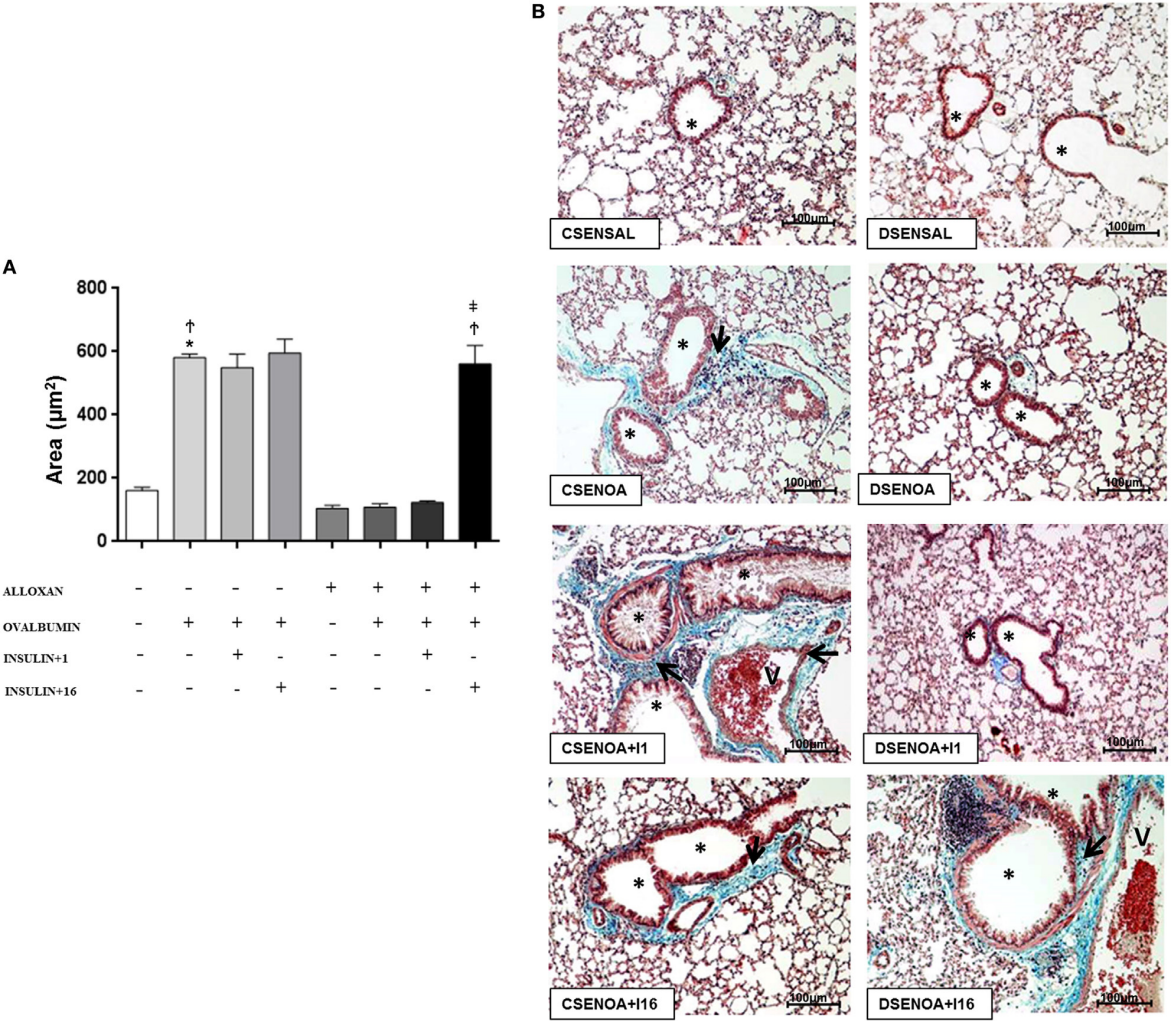
**Collagen secretion: role of insulin**. **(A)** On average, 10 fields were photographed per slide. Quantification was performed using NIS-Elements AR software. Values represent the mean ± SEM leukocytes (*n* = 5 animals/group). **(B)** The microphotographs of lung tissue were obtained from control non-diabetic mice sensitized and instilled with saline (CSENSAL) or sensitized and instilled with ovalbumin (OA) (CSENOA) and treated with single-dose insulin (CSENOA + I1) or treated with consecutive doses of insulin (CSENOA + I16); diabetic mice sensitized and instilled with saline (DSENSAL) or sensitized and instilled with OA (DESENOA) and treated with single-dose insulin (DSENOA + I1) or treated with consecutive doses of insulin (DSENOA + I16). * = bronchus; v = blood vessel; arrow: collagen bars = 100 µm; Masson’s trichrome. Data are representative of five animals per experimental group. Values are shown as the mean ± SEM. *p < 0.01 comparing OA-challenged with saline-challenged groups. ^Ϯ^*p* < 0.01 comparing OA-challenged with diabetic OA-challenged groups. ^‡^*p* < 0.01 comparing diabetic OA-challenged treatment to single-dose insulin. Differences among the groups were tested with one-way analysis of variance followed by Tukey’s *post hoc* test. A *p*-value <0.05 was considered statistically significant (GraphPad Prism version 6.0 for Windows, GraphPad Software, La Jolla, CA, USA).

Compared to SAL-challenged mice, non-diabetic mice presented an increase (3.6-fold) in the collagen secretion of the lung parenchyma after the OA challenge. In contrast, reduced collagen secretions (82%) were observed in diabetic OA-challenged mice compared to those of the control mice. Treatment of diabetic mice with multiple doses of insulin restored collagen secretion in the lung parenchyma (Figure 4).

### Mucus Production: Role of Insulin

**Figure 5:**
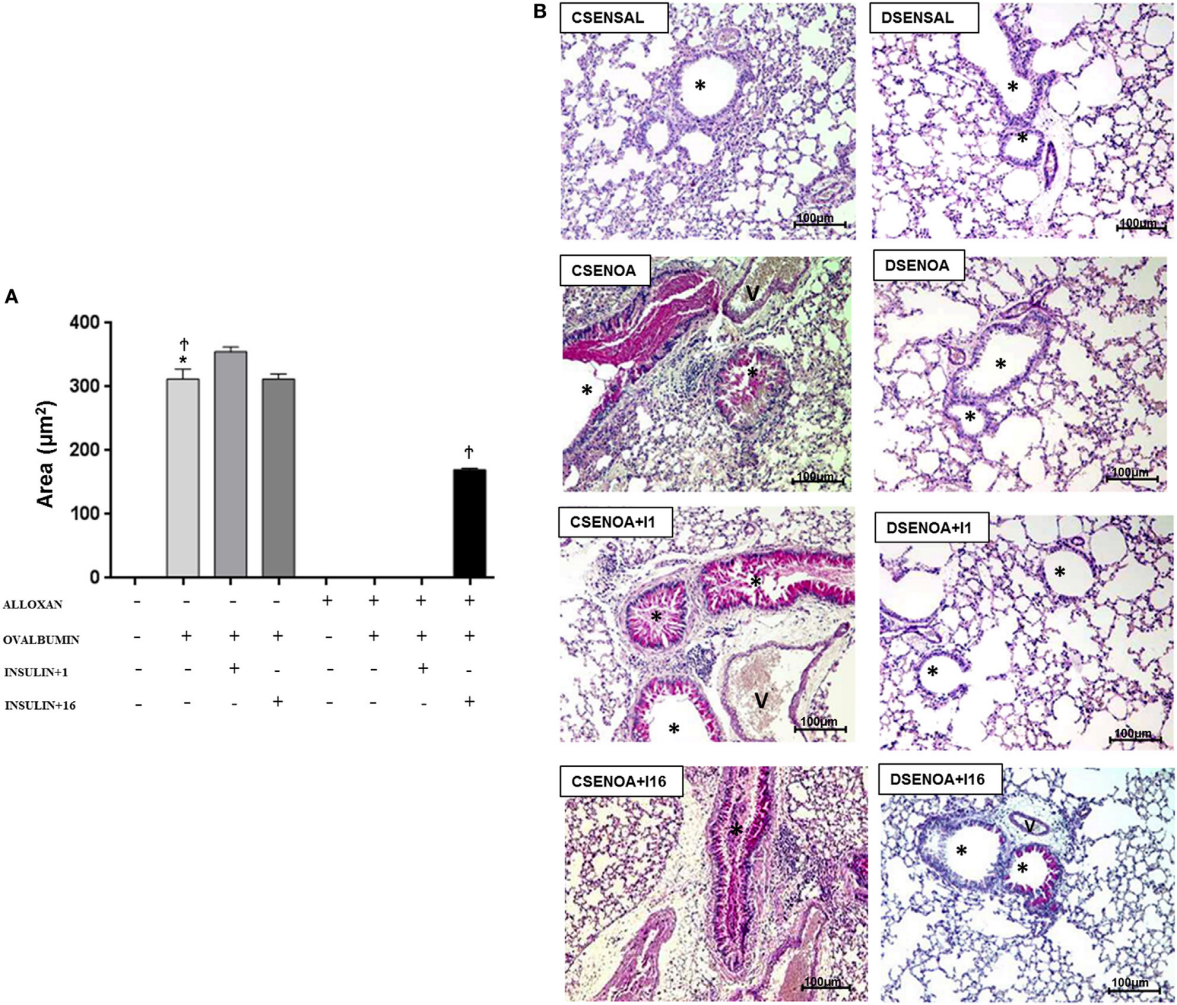
**Mucus production: role of insulin**. **(A)** On average, 10 fields were photographed per slide. Quantification was performed using NIS-Elements AR software. Values represent the mean ± SEM leukocytes (*n* = 5 animals/group). **(B)** The microphotographs of lung tissue were obtained from control non-diabetic mice sensitized and instilled with saline (CSENSAL) or sensitized and instilled with ovalbumin (OA) (CSENOA) and treated with single-dose insulin (CSENOA + I1) or treated with consecutive doses of insulin (CSENOA + I16); diabetic mice sensitized and instilled with saline (DSENSAL) or sensitized and instilled with ovalbumin (OA) (DESENOA) and treated with single-dose insulin (DSENOA + I1) or treated with consecutive doses of insulin (DSENOA + I16). * = bronchus; v = blood vessel; arrow: mucus (bars = 100 µm) PAS. Data are representative of five animals per experimental group. Values are shown as the mean ± SEM. *p < 0.001 comparing OA-challenged with saline-challenged. ^Ϯ^*p* < 0.001 comparing OA-challenged with the diabetic OA-challenged group. ^‡^*p* < 0.001 comparing diabetic OA-challenged treatment to single-dose insulin. Differences among the groups were tested with one-way analysis of variance followed by Tukey’s *post hoc* test. A *p*-value <0.05 was considered statistically significant (GraphPad Prism version 6.0 for Windows, GraphPad Software, La Jolla, CA, USA).

Ovalbumin challenge induced mucus production in the lung parenchyma of non-diabetic mice. In contrast, mucus production was not detected in the lung parenchyma of diabetic OA-challenged mice. Treatment of diabetic mice with multiple dose of insulin restored, at least in part, mucus production in the lung parenchyma (Figure 5).

## Discussion

The data presented here suggest that insulin modulates lung remodeling in allergic inflammatory reactions in the bronchial remodeling phase of a diabetic murine model, restoring: (a) infiltrate inflammation in the BALF; (b) eosinophilia; (c) collagen deposition around the airways; and (d) at least in part, secretion/deposition of mucus within the airways.

This asthma model is widely used and has provided important knowledge about immune and inflammatory mechanisms (18, 19). In general, this model involves sensitization of the mice by intraperitoneal injection of the allergen in combination with adjuvant material, such as Al(OH)_3_, along with allergic exposures at the site (lung). When the animals are evaluated for asthmatic phenotypes, they present hyperresponsiveness of the airways (20), eosinophilia (21), and inflammation with Th2 profile cytokines (22, 23). After repeated exposures to the allergen, structural alterations that culminate in remodeling of the airways can occur (16, 24). This remodeling is characterized by smooth muscle hypertrophy, increased secretion of mucus, and deposition of collagen around the airways, which consequently result in fibrosis (24, 25).

Diabetes mellitus has high morbidity and mortality rates and results in a significant decrease in patient quality of life. One of the leading causes of death in patients with diabetes is renal failure, blindness, lower limb amputation, and cardiovascular disease (26). Additionally, diabetic patients have increased immune dysfunction and are more susceptible to infection (27). In experimental studies, diabetic mice that were infected with *Pseudomonas aeruginosa* had an increase in biofilms in their wounds, and insulin treatment increased the biofilms in the wounds of diabetic mice (28). Moreover, sensitized and OA-challenged diabetic animals exhibited reduced pulmonary inflammatory infiltrate. Treatment of these animals with insulin ameliorated this condition, suggesting that asthma symptoms are suppressed by the diabetic state (13, 29). In addition, insulin treatment amplifies the inflammatory response and hypersensitive reactions, such as tuberculin cutaneous test in rats. Animals treated with insulin, before and after skin challenges, presented gross skin reaction compared to that of untreated animals (30).

In clinical studies, triggering of diabetes mellitus in previously asthmatic patients resulted in an improvement in asthmatic symptoms; treatment of diabetic patients with insulin aggravated asthma, and similar results were observed in non-diabetic asthmatic patients receiving insulin (10, 11). Obesity also aggravates asthma; it was reported that an increase of body mass index (BMI) and/or excess weight may increase the risk of asthma-related hospitalizations or asthma severity (31). The possible underlying mechanisms were extensively discussed by Stephanie (32) and likely include common etiologies and comorbidities between many other factors, such as adipokines, leptin, and proinflammatory cytokines. In addition, to evaluate the anti-inflammatory activity in allergic reactions induced by OA, we measured IL-10 in the BALF of the animals. In our study, IL-10 levels did not differ between groups, which suggest that the phenomenon might be linked to Th1 response polarization of diabetes mellitus type I since Han et al. (33) showed that hyperinsulinemia in obese mice results in a decrease in the production of IL-10 by regulating Treg cells. Moreover, the synergistic contribution of insulin and proinflammatory cytokines to the stimulation of the immune system has been reported. Dror et al. (34) found that insulin stimulates IL-1b secretion by resident macrophages, which could explain the mechanisms by which insulin restored IL-1β levels in our study.

In fact, previous studies by our research group using a model of pulmonary inflammation in the initial phase revealed that insulin modulates the release of cytokines, such as TNF-α and IL-1β, as well as expression of adhesion molecules, such as P and E-selectin, and consequently migration of neutrophils into the lung during the initial phase of the allergic inflammatory reaction (14). The results presented here showed decreased IL-4, IL-5, and IL-13 levels in OA-challenged diabetic mice and that single-dose insulin treatment restored levels of IL-4 and IL-5, although IL-13 was only restored by multiple doses of insulin treatment (16 doses). Although these Th2 profile cytokines play an important role in the eosinophil migration, OA-challenged diabetic mice did not present eosinophilia in both the blood and BALF. Treatment with a single dose of insulin restored eosinophilia parameters in the blood of the animals, but not in BALF, suggesting that 8 h was insufficient time for eosinophil migration to the tissue; however, with multiple doses of insulin, we observed eosinophilia in BALF. In addition, a single dose of insulin did not restore the deposition of mucus and collagen in the airways of OA-challenged diabetic mice. However, multiple doses of insulin restored the deposition of mucus and collagen in the airways, which suggests that appropriate treatment with insulin may modulate cytokine levels, cell migration, eosinophilia, and mucus and collagen deposition in lung remodeling in the murine asthma model.

The data presented here suggest that insulin regulates lung remodeling in an experimental model of allergic airway inflammation in diabetic mice by controlling cytokines, cell migration, collagen deposition, and mucus secretion into the lungs.

## Ethics Statement

This study was carried out in strict accordance with the principles and guidelines adopted by the Brazilian National Council for the Control of Animal Experimentation (CONCEA) and approved by the Ethical Committee on Animal Use (CEUA) of the Faculty of Pharmaceutical Sciences (FCF) of University São Paulo (Permit Number: CEUA/FCF/340).

## Author Contributions

SF and JM conceived and designed the experiments; wrote the paper with the assistance of all the authors. SF, FN, and FC performed the experiments. SF, FN, and JM analyzed the data. JM contributed reagents/materials/analysis tools.

## Conflict of Interest Statement

The authors declare that the research was conducted in the absence of any commercial or financial relationships that could be construed as a potential conflict of interest.

## References

[1] Sociedade Brasileira de Pneumonia e Tisiologia. Diretrizes da Sociedade Brasileira de Pneumologia e Tisiologia. J Bras Pneumol (2012) 38(1):S1–46.

[2] Galli SJ Tsai M Piliponsky AM The development of allergic inflammation. Nature (2008) 454(7203):445–54. 10.1038/nature07204 18650915 PMC3573758

[3] Deckers J Madeira FB Hammad H Innate immune cells in asthma. Trends Immunol (2013) 34(11):540–7. 10.1016/j.it.2013.08.004 24035576

[4] Holgate ST Pathogenesis of asthma. Clin Exp Allergy (2008) 38(6):872–97. 10.1111/j.1365-2222.2008.02971.x 18498538

[5] Schofield M Calhoun KH Immunology of asthma. Otolaryngol Clin N Am (2011) 44:591–601. 10.1016/j.otc.2011.03.002 21621047

[6] Yang IV Schwartz DA Epigenetic mechanisms and the development of asthma. J Clin Immunol (2012) 130(6):1243–55. 10.1016/j.jaci.2012.07.052 PMC3518374 23026498

[7] Pascual RM Peters SP. Airway remodeling contributes to the progressive loss of lung function in asthma: an overview. J Allergy Clin Immunol (2005) 116(3):477–86. 10.1016/j.jaci.2005.07.011 16159612

[8] Holgate ST Polosa R Treatment strategies for allergy and asthma. Nat Rev Immunol (2008) 8:218–30. 10.1038/nri2262 18274559

[9] GINA. Global Initiative for Asthma. (2016). Available from: http://ginasthma.org/

[10] Helander E Asthma and diabetes. Acta Med Scand (1958) 162:165–74. 10.1111/j.0954-6820.1958.tb01762.x 13605593

[11] Douek IF Leech NJ Gillmor HA Bingley PJ Gale EAM Children with type-1 diabetes and their unaffected siblings have fewer symptoms of asthma. Lancet (1999) 353:1850 10.1016/S0140-6736(99)00988-5 10359413

[12] Vianna ESO Garcia-Leme J. Allergen-induced airway inflammation in rats. Role of insulin. Am J Respir Crit Care Med (1995) 151:809–14. 10.1164/ajrccm.151.3.7881676 7881676

[13] Cavalher-Machado SC De Lima WT Damazo AS Frias de Carvalho V Martins MA Silva PMR Down-regulation of mast cell activation and airway reactivity in diabetic rats: role of insulin. Eur Respir J (2004) 24:552–8. 10.1183/09031936.04.00130803 15459132

[14] Martins JO Campos CAL Cruz JWMC Manzolli S Alves VAF Vianna EO Insulin modulates cytokine release and selectin expression in the early phase of allergic airway inflammation in diabetic rats. BMC Pulm Med (2010) 10:39. 10.1186/1471-2466-10-39 20667094 PMC2916891

[15] Spiller F Carlos D Souto FO de Freitas A Soares FS Vieira SM α1-Acid glycoprotein decreases neutrophil migration and increases susceptibility to sepsis in diabetic mice. Diabetes (2012) 61:1584–1584. 10.2337/db11-0825 22415874 PMC3357278

[16] Stumm CL Wettlauer SH Jancar S Peters-Golden M. Airway remodeling in murine asthma correlates with a defect in PGE_2_ synthesis by lung fibroblasts. Am J Physiol Lung Cell Mol Physiol (2011) 301(5):L636–44. 10.1152/ajplung.00158.2011 21873451 PMC3213985

[17] Di Petta A Greco OE Lopes FFTQS Martins MA Capellozi VL Moreira LFP Insulin modulates inflammatory and repair responses to elastase-induced emphysema in diabetic rats. Int J Exp Pathol (2011) 92:392–9. 10.1111/j.1365-2613.2011.00787.x 21950537 PMC3248075

[18] Emala C Hirshman C Animal models of bronchial hyperreactivity. Monogr Allergy (1996) 33:35–52. 8930925

[19] Herz U Lumpp U Da Palma JC Enssle K Takatsu K Schonoy N The relevance of murine animal models to study the development of allergic bronchial asthma. Immunol Cell Biol (1996) 74:209–17. 10.1038/icb.1996.30 8724012

[20] Barnes PJ. New drugs for asthma. Nat Rev Drug Discov (2004) 3:831–44. 10.1038/nrd1524 15459674

[21] Kay AB. The role of eosinophils in the pathogenesis of asthma. Trends Mol Med (2005) 11:148–52. 10.1016/j.molmed.2005.02.002 15823751

[22] Pauwels RA Brussellee GJ Kips JC. Cytokine manipulation in animal models of asthma. Am J Respir Crit Care Med (1997) 156:S78–81. 10.1164/ajrccm.156.4.12-tac-1 9351584

[23] Manise M Holtappels G Crombruggen KV Schleich F Bachertz C Louis R. Sputum IgE and cytokines in asthma: relationship with sputum cellular profile. PLoS One (2013) 8(3):e58388. 10.1371/journal.pone.0058388 23555579 PMC3608646

[24] Stumm CL Halcsik E Landgraf RG Camara NOS Sogayar MC Jancar S Lung remodeling in a mouse model of asthma involves a balance between TGF-B1 and BMP-7. PLoS One (2014) 9(4):e95959 10.1371/journal.pone.0095959 24781156 PMC4004563

[25] Boulet L-P Sterk PJ Airway remodeling: the future. Eur Respir J (2007) 30:831–4. 10.1183/09031936.00110107 17978153

[26] International Diabetes Federation. IDF Diabetes Atlas. 7th ed Brussels: International Diabetes Federation (2015).

[27] Lerco MM Spadella CT Machado JLM Schellini SA Padovani CR Caracterização de um modelo experimental de *Diabetes mellitus*, induzido pela aloxana em ratos. Acta Cirúrgica Brasileira (2003) 18(2):132–42. 10.1590/S0102-86502003000200010

[28] Watters C Everett A Haley C Clinton A Rumbaugha KP Insulin treatment modulates the host immune system to enhance *Pseudomonas aeruginosa* wound biofilms. Infect Immun (2014) 82:92–100. 10.1128/IAI.00651-13 24126517 PMC3911840

[29] Singh S Prakash YS Linneberg A Agrawal A Insulin and the lung connecting asthma and metabolic syndrome. J Allergy (2013) 2013:8 10.1155/2013/627384 PMC3800560 24204385

[30] Thompson GE Enhancing effect of insulin on the tuberculin reaction in the albino rat. Nature (1967) 215:748–9. 10.1038/215748a0 6059546

[31] Mossem DM Schatz M Magid DJ Camargo CA The relationship between obesity and asthma severity and control in adults. J Allergy Clin Immunol (2008) 122:507–11. 10.1016/j.jaci.2008.06.024 18774387

[32] Shore SA. Obesity and asthma: lessons from animal models. J Appl Physiol (2007) 102:516–28. 10.1152/japplphysiol.00847.2006 17053103

[33] Han JM Patterson SJ Speck M Ehses JA Levings MK. Insulin inhibits IL-10-mediated regulatory T cell function: implications for obesity. J Immunol (2014) 192:623–9. 10.4049/jimmunol.1302181 24323581

[34] Dror E Dalmas E Meire DT Wuesst S Thévenet J Thienel C Postprandial macrophage-derived Il-1β stimulates insulin, and both synergistically promote glucose disposal and inflammation. Nat Immunol (2017) 18:283–92. 10.1038/ni.3659 28092375

